# A Sign of Consumption

**Published:** 1881-12

**Authors:** 


**It is not known as generally as it ought to be, that consumption, that disease known as "common consumption of the lungs," called by many a decline," begins with a regular hacking cough in the morning, or on getting up. In fact it is so slight at first, that it scarcely amounts to a cough; it is a mere "hem" or effort to clear the throat of something which seems to excite a little tickling sensation there.**

